# Effect of butyrate‐producing enterobacteria on advanced hepatocellular carcinoma treatment with atezolizumab and bevacizumab

**DOI:** 10.1002/cam4.6416

**Published:** 2023-08-11

**Authors:** Kazuhiro Nouso, Shohei Shiota, Rio Fujita, Akiko Wakuta, Kazuya Kariyama, Atsushi Hiraoka, Masanori Atsukawa, Joji Tani, Toshifumi Tada, Shinichiro Nakamura, Kazuto Tajiri, Masaki Kaibori, Masashi Hirooka, Ei Itobayashi, Satoru Kakizaki, Atsushi Naganuma, Toru Ishikawa, Takeshi Hatanaka, Shinya Fukunishi, Kunihiko Tsuji, Kazuhito Kawata, Koichi Takaguchi, Akemi Tsutsui, Chikara Ogawa, Hironori Ochi, Yutaka Yata, Hidekatsu Kuroda, Hiroko Iijima, Tomomitsu Matono, Noritomo Shimada, Satoshi Yasuda, Hidenori Toyoda, Takashi Kumada, the Real‐life Practice Experts for HCC (RELPEC) Study Group and HCC 48 Group (hepatocellular carcinoma experts from 48 clinics in Japan)

**Affiliations:** ^1^ Department of Gastroenterology Okayama City Hospital Okayama Japan; ^2^ Gastroenterology Center Ehime Prefectural Central Hospital Matsuyama Japan; ^3^ Division of Gastroenterology and Hepatology, Department of Internal Medicine Nippon Medical School Tokyo Japan; ^4^ Department of Gastroenterology and Hepatology Kagawa University Takamatsu Japan; ^5^ Department of Internal Medicine Japanese Red Cross Society Himeji Hospital Himeji Japan; ^6^ Department of Gastroenterology Toyama University Hospital Toyama Japan; ^7^ Department of Surgery Kansai Medical University Hirakata Japan; ^8^ Department of Gastroenterology and Metabology Ehime University Graduate School of Medicine Toon Japan; ^9^ Department of Gastroenterology Asahi General Hospital Asahi Japan; ^10^ Department of Clinical Research National Hospital Organization Takasaki General Medical Center Takasaki Japan; ^11^ Department of Gastroenterology National Hospital Organization Takasaki General Medical Center Takasaki Japan; ^12^ Department of Gastroenterology Saiseikai Niigata Hospital Niigata Japan; ^13^ Department of Gastroenterology Gunma Saiseikai Maebashi Hospital Maebashi Japan; ^14^ Department of Gastroenterology Osaka Medical and Pharmaceutical University Osaka Japan; ^15^ Gastroenterology Center, Teine Keijinkai Hospital Sapporo Japan; ^16^ Hepatology Division, Department of Internal Medicine II Hamamatsu University School of Medicine Hamamatsu Japan; ^17^ Department of Hepatology Kagawa Prefectural Central Hospital Takamatsu Japan; ^18^ Department of Gastroenterology Japanese Red Cross Takamatsu Hospital Takamatsu Japan; ^19^ Center for Liver‐Biliary‐Pancreatic Disease Matsuyama Red Cross Hospital Matsuyama Japan; ^20^ Department of Gastroenterology Hanwa Memorial Hospital Osaka Japan; ^21^ Department of Gastroenterology Iwate Medical University Iwate Japan; ^22^ Division of Gastroenterology and Hepatobiliary and Pancreatic Diseases, Department of Internal Medicine Hyogo Medical University Himeji Japan; ^23^ Department of Gastroenterology and Hepatology St. Mary's Hospital Himeji Japan; ^24^ Division of Gastroenterology and Hepatology Otakanomori Hospital Kashiwa Japan; ^25^ Department of Gastroenterology and Hepatology Ogaki Municipal Hospital Gifu Japan; ^26^ Department of Nursing Gifu Kyoritsu University Ogaki Japan

**Keywords:** atezolizumab, bevacizumab, butyrate‐producing enterobacteria, hepatocellular carcinoma

## Abstract

**Aim:**

Multiple studies have revealed the correlation between gut microbiome and the response to checkpoint inhibitors (CPIs) in patients with cancer, and oral administration of butyrate‐producing enterobacteria has been reported to enhance the efficacy of CPIs. However, the effects of enterobacteria on patients with hepatocellular carcinoma (HCC) are not well understood.

**Methods:**

In this retrospective multicenter study, we enrolled 747 patients with advanced HCC, treated with atezolizumab and bevacizumab combination therapy. Tumor response, survival, and adverse effects were compared between 99 patients who ingested drugs containing butyric acid‐producing enterobacteria (butyric acid group) and the remaining patients (control group).

**Results:**

Objective response and disease control rates in butyric acid group (29.7% and 77.8%, respectively) were higher than those in the control group (26.4% and 72.7%, respectively). However, the differences were not statistically significant (*p* = 0.543 and *p* = 0.222, respectively). No difference in median survival time was observed between the two groups (20.0 months and 21.4 months, respectively; *p* = 0.789), even after matching the backgrounds of the patients with propensity scores (*p* = 0.714). No adverse effects occurred upon the administration of butyrate‐producing bacteria. However, proteinuria (41.4% vs. 30.9%; *p* = 0.041), fever (17.2% vs. 10.2%, *p* = 0.036), and diarrhea (15.2% vs. 6.2%; *p* = 0.001) occurred more frequently in the butyric acid group.

**Conclusion:**

Butyrate‐producing bacteria does not enhance the efficacy of atezolizumab–bevacizumab combination therapy in patients with HCC.

## 1 INTRODUCTION

Hepatocellular carcinoma (HCC) is the third leading cause of cancer deaths worldwide.^1^ Although early detection has been achieved in some regions by screening patients with hepatitis virus infection, many patients have been found to be at an advanced stage.^2^ Recently, several systemic chemotherapies have been used in clinical practice^3^, ^4^ including the combination of immunotherapy with atezolizumab and bevacizumab, which showed the best results. Therefore, many guidelines regarding HCC treatment recommend it as the first‐line therapy for advanced HCC.^4^, ^5^, ^6^, ^7^

Enterobacteria is associated with the pathophysiology and pathogenesis of many diseases.^8^ Previous studies have reported the link between gut microbiome and the prognosis of multiple cancers^9^, ^10^, ^11^, ^12^ and the beneficial effects of supplementing cancer patients with butyrate‐producing bacteria.^13^, ^14^, ^15^ Dizman et al. reported that the administration of CBM588, which contains butyric acid‐producing bacteria, prolonged the progression‐free survival of patients with metastatic renal cell carcinoma treated with nivolumab and ipilimumab, which are checkpoint inhibitors (CPIs).^15^ However, the correlation between enterobacteria and the effects of chemotherapy, including CPIs, in patients with HCC is still obscure.

In this study, we examined the effects of butyric acid‐producing enterobacteria on patients with advanced HCC treated with atezolizumab and bevacizumab.

## 2 PATIENTS AND METHODS

We enrolled 747 patients with advanced HCC, treated with atezolizumab and bevacizumab at 25 different hospitals in Japan between November 2020 and January 2023 (Kansai Medical University, Kagawa University, Takasaki General Medical Center, Teine Keijinkai Hospital, Ehime Prefectural Central Hospital, Saiseikai Niigata Hospital, Osaka Medical and Pharmaceutical University, Hamamatsu University School of Medicine, University of Toyama, Asahi General Hospital, Ehime University, Kagawa Prefectural Central Hospital, Takamatsu Red Cross Hospital, Gunma Saiseikai Maebashi Hospital, Matsuyama Red Cross Hospital, Nippon Medical School Hospital, Nippon Medical School Chibahokusoh Hospital, Okayama City Hospital, Hanwa Memorial Hospital, Iwate Medical University, Hyogo Medical University, Otakanomori Hospital, Himeji St. Mary's Hospital, Ogaki Municipal Hospital, and Himeji Red Cross Hospital). The final observation date was in January 2023.

All patients provided consent to review their clinical records for this study. The study protocol conformed to the tenets of the Declaration of Helsinki and was approved by the Institutional Ethics Committee of Ehime Prefectural Central Hospital (IRB # 30–66) (UMIN000043219) and each participating institution.

### 2.1 Treatment

The patients received 1200 mg of atezolizumab and 15 mg/kg of body weight of bevacizumab intravenously once every 3 weeks, according to the manufacturer's instructions. Therapy was discontinued if the tumor progressed or if unacceptable adverse effects (AEs) were determined by each physician based on the patient's age and status. Moreover, AEs were managed by interrupting treatment or reducing the dose according to the instructions provided by the supplier. Patients who ingested drugs containing butyric acid‐producing enterobacteria (MIYA‐BM, MIYARISAN Pharmaceutical CO., LTD. or BIO‐THREE, TOA Pharmaceuticals CO., LTD.) during treatment were referred to as the butyric acid group and the others as the control group. Tumor response was assessed via computed tomography or magnetic resonance imaging every 2–3 months, according to the Response Evaluation Criteria in Solid Tumors version 1.1.^16^

### 2.2 Statistical analysis

Baseline characteristics are presented as medians and ranges. Continuous variables were compared via the Wilcoxon rank‐sum test and categorical values were analyzed via the chi‐squared test. The prognosis was analyzed via the Kaplan–Meier method and the log‐rank test. Propensity score matching was performed for age, sex, albumin‐bilirubin (ALBI) score, Barcelona clinic liver cancer (BCLC) stage, cause of HCC, and the first‐line treatment group as the matching variables. The caliper of the analysis was 0.3, and 1 × 2 matching was performed. All significance tests were two‐sided; *p*‐values <0.05 were considered statistically significant. All analyses were performed via the JMP software (version 16.0; SAS Institute Japan Ltd., Tokyo, Japan), except for the survival analysis, for which we used EZR (Saitama Medical Center, Jichi Medical University, Saitama, Japan), a graphical user interface for R (The R Foundation for Statistical Computing, Vienna, Austria).

## 3 RESULTS

### 3.1 Patients' characteristics

Of the 747 HCC patients treated with atezolizumab and bevacizumab, 99 ingested drugs containing butyric acid‐producing enterobacteria (butyric acid group), and most of them consumed MIYA‐BM (84 patients; 84.8%). Many patients had been prescribed the drug by previous doctors, and the specific reason for its administration in those patients was unclear, possibly due to irritable bowel syndrome. However, 13 patients (13.1%) experienced diarrhea as an adverse event of chemotherapy.

The median age of the patients was 74 years in both the butyric acid and control groups, and the cause of HCC was nonviral in 45.5% and 50.9% of patients in the butyric acid and control groups, respectively (Table 1). Atezolizumab and bevacizumab were used as first‐line systemic therapies in 58.6% of the patients in the butyric acid group. However, the percentage was significantly lower than that in the control group (68.7%; *p* = 0.046). ALBI score in butyric acid group was significantly higher than that in control group (−2.23 vs. ‐2.43; *p* = 0.001). No differences were observed in other variables between the butyric acid and control groups.

**TABLE 1:** Characteristics of the patients.

Variable	Butyric acid group (*n* = 99)	Control group (*n* = 648)	*p*‐value
Age, year	74 (42–90)	74 (27–94)	0.541
Male sex	81 (81.8%)	519 (80.1%)	0.687
Cause of hepatocellular carcinoma			0.138
Hepatitis B	11 (11.1%)	109 (16.8%)	
Hepatitis C	43 (43.4%)	210 (32.4%)	
Nonviral	45 (45.5%)	330 (50.9%)	
ECOG performance status, 0/1/2‐	88/10/1	518/107/23	0.089
Use in first‐line systemic therapy	58 (58.6%)	445 (68.7%)	0.046
BCLC stage, A/B/C/D	9/39/59/1	54/225/353/16	0.649
Intrahepatic tumor size (≧5 cm)	34 (34.3%)	219 (33.8%)	0.947
Intrahepatic tumor number (≧5)	44 (44.4%)	293 (45.2%)	0.992
Portal vein invasion (1/2/3/4)	6/5/8/2	20/38/36/19	0.460
Presence of major vascular invasion	24 (24.2%)	122 (18.8%)	0.216
Presence of extrahepatic spread	27 (37.5%)	211 (32.6%)	0.292
ALBI score	−2.23 (−3.35 to −1.31)	−2.43 (−3.50 to −0.81)	0.001
Child–Pugh stage A	86 (86.9%)	579 (89.4%)	0.461

*Note*: Values are indicated as median (range) unless otherwise noted.

Abbreviations: ALBI, albumin‐bilirubin; BCLC, Barcelona clinic liver cancer; ECOG, Eastern Cooperative Oncology Group.

### 3.2 Effect of butyric acid‐producing enterobacteria on tumor response

The best responses in the butyric acid and control groups are shown in Table 2. Objective response and disease control rates in butyric acid group (29.7% and 77.8%, respectively) were higher than those in the control group (26.4% and 72.7%, respectively). However, the differences were not statistically significant (*p* = 0.543 and *p* = 0.222, respectively). No statistical difference in objective response rate and disease control rate was observed between the butyric acid group and the control group, irrespective of the line of treatment (Table 3).

**TABLE 2:** Best response evaluated by RECIST.

	CR	PR	SD	PD	NE	ORR	DCR
Butyric acid group	7 (7.1%)	22 (22.2%)	48 (48.5%)	16 (16.2%)	6 (6.1%)	29* (29.3%)	77** (77.8%)
Control group	21 (3.2%)	150 (23.1%)	295 (45.5%)	127 (19.6%)	55 (8.5%)	171* (26.4%)	466** (71.9%)
Total	28 (3.8%)	172 (23.0%)	343 (45.9%)	143 (19.1%)	61 (8.2%)	200 (26.8%)	543 (72.7%)

Abbreviations: CR, complete response; DCR, disease control rate; PD, progressive disease; PR, partial response; NE, not evaluable; ORR, objective response rate; SD, stable disease.

* *p* = 0.543

** *p* = 0.222.

**TABLE 3:** Best response in different line of treatment.

	First‐line treatment		Later‐line treatment	
	ORR	DCR	ORR	DCR
Butyric acid group	14 (24.1%)	44 (75.9%)	15 (36.6%)	33 (80.5%)
Control group	125 (28.1%)	314 (70.6%)	46 (22.7%)	152 (74.9%)
*p*‐value	0.521	0.401	0.060	0.444

Abbreviations: DCR, disease control rate; ORR, objective response ratekm.

### 3.3 Effect of butyric acid‐producing enterobacteria on survival

**FIGURE 1:**
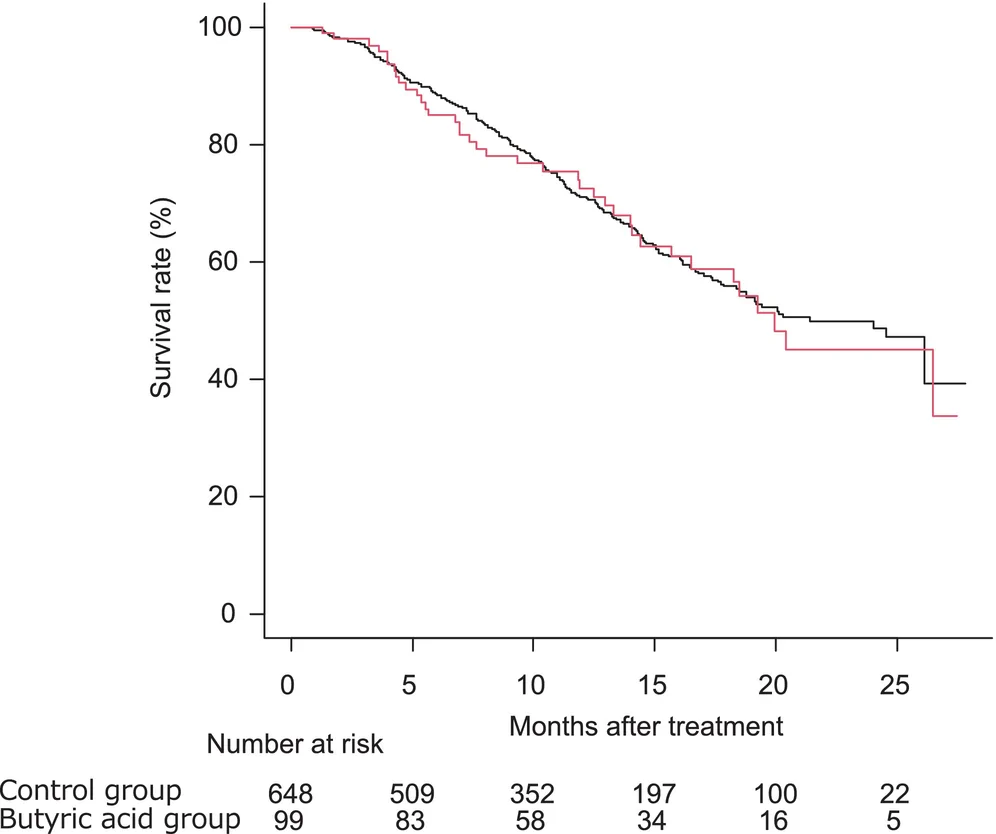
Overall survival of patients with hepatocellular carcinoma treated with atezolizumab and bevacizumab combination therapy. Median survival times in butyric acid group (red) and control group (black) were 20.0 months and 21.4 months, respectively, and the difference was not statistically significant (*p* = 0.789).

**FIGURE 2:**
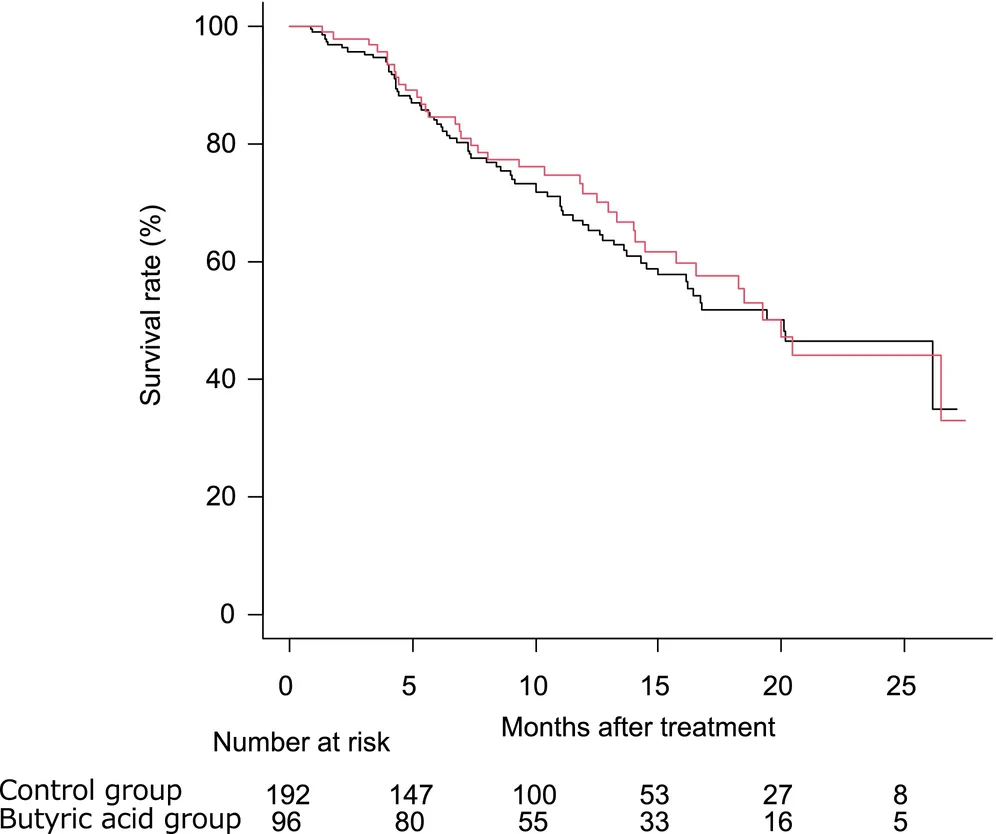
Comparison of overall survival after propensity score matching. Propensity score matching was performed for age, sex, albumin‐bilirubin score, Barcelona clinic liver cancer stage, cause of hepatocellular carcinoma, and the first‐line use or not. The median survival time in the butyric acid group (20.0 months, red) was not significantly different from that in the control group (20.1 months, black; *p* = 0.714).

Median survival times in butyric acid group and control group were 20.0 months and 21.4 months, respectively (Figure 1), and the difference was not statistically significant (*p* = 0.789). Propensity score matching was performed for age, sex, ALBI score, BCLC stage, cause of HCC, and the first‐line treatment group, and the survival times of the two groups were compared to avoid the effect of differences in background factors (Figure 2). The median survival time in the butyric acid group (20.0 months) was not significantly different from that in the control group (20.1 months; *p* = 0.714).

We did not observe any differences in OS even when examining patients undergoing first‐line treatment (*p* = 0.394).

### 3.4 Effect of butyric acid‐producing enterobacteria on AEs

The most prevalent AE was proteinuria, followed by fatigue (Table 4). Both the butyric acid and control groups showed similar patterns of AEs. However, proteinuria (41.4% vs. 30.9%; *p* = 0.041), fever (17.2% vs. 10.2%; *p* = 0.036), and diarrhea (15.2% vs. 6.2%; *p* = 0.001) were more frequently observed in the butyric acid group than in the control group. No unpredictable AEs were observed in either group.

**TABLE 4:** Adverse effects.

Adverse effect	Butyric acid group (*n* = 99)		Control group (*n* = 648)		*p*‐value^a^
	Any grade	Grade 3/4	Any grade	Grade 3/4	
Proteinuria	41 (41.4%)	7 (7.1%)	200 (30.9%)	63 (9.7%)	0.041
Fatigue	31 (31.3%)	4 (4.0%)	144 (22.2%)	14 (2.2%)	0.050
Appetite loss	20 (20.2%)	3 (3.0%)	135 (20.1%)	19 (2.9%)	0.862
Liver dysfunction	20 (20.2%)	6 (6.1%)	98 (15.1%)	22 (3.4%)	0.206
Hypertension	16 (16.2%)	4 (4.0%)	101 (15.6%)	29 (4.5%)	0.908
Exanthema	16 (16.2%)	2 (2.0%)	74 (11.4%)	12 (1.9%)	0.186
Edema/ascites	8 (8.1%)	4 (4.0%)	80 (12.3%)	30 (4.6%)	0.232
Fever	17 (17.2%)	1 (1.0%)	66 (10.2%)	9 (1.4%)	0.036
Diarrhea	15 (15.2%)	5 (5.1%)	40 (6.2%)	4 (0.6%)	0.001

^a^ *p*‐value indicates the difference in adverse effects of any grade between the butyric acid group and control group.

## 4 DISCUSSION

Butyrate‐producing enterobacteria are bifidogenic and promote dendritic cell function and T‐cell recruitment. This leads to an increased efficacy of CPIs.^17^ Therefore, we examined the effect of butyrate‐producing bacteria on the prognosis of patients with HCC, treated with atezolizumab and bevacizumab combination therapy. We did not observe any benefits of butyrate‐producing bacteria on patients with HCC, unlike the case in patients with non‐small cell lung cancer or metastatic renal cancer treated with CPIs.^13^, ^14^, ^15^ The tumor response and overall survival did not differ between the butyric acid and control groups, even after propensity matching the background of the patients. In addition, AEs such as proteinuria, fever, and diarrhea were more frequently observed in the butyric acid group.

There are several possible reasons why we did not observe beneficial effects of butyrate‐producing bacteria. First, the studies mentioned above reported the benefits of drugs that block PD1/PDL1 alone or also block CTLA4 for treating cancers, regardless of the tumor type.^13^, ^14^, ^15^ These drugs are CPIs. Therefore, the effect of atezolizumab and bevacizumab combination therapy might differ because the latter is an anti‐vascular endothelial growth factor (VEGF) antibody. Certain species of *Bifirobacterium* have been reported to lack a response to VEGF inhibition. Therefore, combination therapy with a VEGF inhibitor might reduce the effect of butyrate‐producing bacteria.^13^

Second, patients in the butyric acid group might have had some physical deconditioning that weakened the effects of atezolizumab and bevacizumab. Although the difference in AEs between the butyric acid and control groups has not been reported in a randomized study,^13^ the incidence of AEs, including diarrhea, was more frequent in the butyric acid group in this study. These results suggest the presence of physical deconditioning that increased AEs in the butyric acid group, although the precise reasons for taking the drugs for each patient are unknown.

Another possibility is that we did not restrict the consumption of bacteria‐fortified foods, such as yogurt and other supplements, that might influence microbiome composition, which are usually not allowed to be consumed by control groups in randomized studies.^13^, ^15^ The absence of these restrictions was considered to diminish the difference between the results in butyric acid and control groups. Furthermore, the frequent consumption of fermented foods such as Natto and Miso in Japan might also reduce the difference as they are bifidogenic.^18^ Therefore, a prospective study with restrictions on food intake is mandatory to determine the actual effects of butyrate‐producing bacteria. The retrospective nature of this study is a major limitation.

The effect of butyrate‐producing enterobacteria may vary depending on the dosing period. In fact, three patients took the drug for less than 1 month due to transient diarrhea. However, in the butyric acid group, the majority of patients (96 patients) took the drug for over a month, making the impact of the three patients negligible.

In addition to butyrate‐producing enterobacteria, antibiotics are also known to alter the gut microbiome. Although there is a report that demonstrated the negative impact of antibiotics on the efficacy of atezolizumab and bevacizumab combination therapy in patients with HCC,^19^ we did not observe any effects of antibiotics or proton pump inhibitors.^20^ This controversial effect could serve as another focus for future prospective studies.

In conclusion, we did not observe any beneficial effect of butyric acid‐producing enterobacteria in patients with HCC treated with atezolizumab and bevacizumab combination therapy. However, recently, a combination therapy with durvalumab and tremelimumab, both of which are CPIs, has been developed for the treatment of HCC.^21^ Therefore, butyric acid‐producing enterobacteria may be more useful. Further prospective studies are required to confirm this hypothesis.

## AUTHOR CONTRIBUTIONS

**Kazuhiro Nouso:** Conceptualization (lead); data curation (lead); formal analysis (lead); investigation (lead); methodology (lead); writing – original draft (lead); writing – review and editing (lead). **Shohei Shiota:** Data curation (equal); resources (equal). **Rio Fujita:** Data curation (equal); resources (equal). **Akiko Wakuta:** Data curation (equal); resources (equal). **Kazuya Kariyama:** Data curation (equal); resources (equal). **Atsushi Hiraoka:** Data curation (equal); resources (equal). **Masanori Atsukawa:** Data curation (equal); resources (equal). **Joji Tani:** Data curation (equal); resources (equal). **Toshifumi Tada:** Data curation (equal); resources (equal). **Shinichiro Nakamura:** Data curation (equal); resources (equal). **Kazuto Tajiri:** Data curation (equal); resources (equal). **Masaki Kaibori:** Data curation (equal); resources (equal). **Masashi Hirooka:** Data curation (equal); resources (equal). **Ei Itobayashi:** Data curation (equal); resources (equal). **Kakizaki Satoru:** Data curation (equal); resources (equal). **Atsushi Naganuma:** Data curation (equal); resources (equal). **Toru Ishikawa:** Data curation (equal); resources (equal). **Takeshi Hatanaka:** Data curation (equal); resources (equal). **Shinya Fukunishi:** Data curation (equal); resources (equal). **Kunihiko Tsuji:** Data curation (equal); resources (equal). **Kazuhito Kawata:** Data curation (equal); resources (equal). **Koichi Takaguchi:** Data curation (equal); resources (equal). **Akemi Tsutsui:** Data curation (equal); resources (equal). **Chikara Ogawa:** Data curation (equal); resources (equal). **Hironori Ochi:** Data curation (equal); resources (equal). **Yutaka Yata:** Data curation (equal); resources (equal). **Hidekatsu Kuroda:** Data curation (equal); resources (equal). **Hiroko Iijima:** Data curation (equal); resources (equal). **Tomomitsu Matono:** Data curation (equal); resources (equal). **Noritomo Shimada:** Data curation (equal); resources (equal). **Satoshi Yasuda:** Data curation (equal); resources (equal). **Hidenori Toyoda:** Data curation (equal); resources (equal). **Takashi Kumada:** Supervision (equal).

## FUNDING INFORMATION

This research received no external funding. Authors A.H. and T.T. received lecture fee from Chugai.

## ETHICS STATEMENT

*Approval of the research protocol*: The study was approved by the Institutional Ethics Committee of Ehime Prefectural Central Hospital (IRB # 30–66) (UMIN000043219) and each participating institution.

## INFORMED CONSENT

All patients provided consent to review their clinical records for this study.

## Data Availability

Data sharing is not applicable to this article as no new data were created or analyzed in this study.
